# Why underserved patients do not consult their general practitioner for depression: results of a qualitative and a quantitative survey at a free outpatient clinic in Paris, France

**DOI:** 10.1186/s12875-015-0273-2

**Published:** 2015-05-08

**Authors:** Claire Rondet, Isabelle Parizot, Jean Sebastien Cadwallader, Jacques Lebas, Pierre Chauvin

**Affiliations:** INSERM, UMR_S 1136, Pierre Louis Institute of Epidemiology and Public Health, Department of Social Epidemiology, F-75013 Paris, France; Sorbonne Universités, UPMC Univ Paris 06, UMR_S 1136, F-75013 Paris, France; Sorbonne Universités, UPMC Univ Paris 06, School of Medicine, Department of General Practice, F-75012 Paris, France; CNRS, UMR 8097, Centre Maurice Halbwachs, Research Team on Social Inequalities, F-75014 Paris, France; INSERM, U669, Paris Sud Innovation Group In Adolescent Mental Health, Cochin Hospital, Paris, France; AP-HP, Hôpital Saint-Antoine, Policlinique Baudelaire, Paris, F-75012 France

**Keywords:** Depression, Primary care, Social inequities, France

## Abstract

**Background:**

The prevalence of depression in the general population is 5 to 10% but can exceed 50% in the most socially vulnerable populations. The perceptions of this disease are widely described in the literature, but no research has been carried out in France to explain the reasons for not consulting a general practitioner during a depressive episode, particularly in people in the most precarious situations. The objective of this study was to describe the reasons for not seeking primary care during a depressive episode in a socially vulnerable population.

**Methods:**

An exploratory sequential design with a preliminary qualitative study using a phenomenological approach. Subsequently, themes that emerged from the qualitative analysis were used in a questionnaire administered in a cross-sectional observational study at a free outpatient clinic in Paris in 2010. Lastly, a logistic regression analysis was performed.

**Results:**

The qualitative analysis revealed four aspects that explain the non-consulting of a general practitioner during a depressive episode: the negative perception of treatment, the negative perception of the disease, the importance of the social environment, and the doctor-patient relationship. The quantitative analysis showed that close to 60% of the patients who visited the free clinic were depressed and that only half of them had talked with a care provider. The results of the statistical analysis are in line with those of the qualitative analysis, since the most common reasons for not seeing a general practitioner were the negative perception of the disease (especially among the men and foreigners) and its treatments (more often among the men and French nationals).

**Conclusions:**

Close to 50% of the depressed individuals did not seek primary care during a depressive episode, and close to 80% of them would have liked their mental health to be discussed more often by a health professional. Better information on depression and its treatments, and more-systematic screening by primary care personnel would improve the treatment of depressed patients, especially those in the most precarious situations.

## Background

In France, the prevalence of depression in the general population is estimated at between 5 and 12% [1-3] and is significantly higher in underserved populations [4,5]. The perceptions of this disease and its treatments, the social factors associated with these perceptions and their impact on adherence to care and therapeutic compliance have been widely described in the international literature [6-12]. However, few studies have examined the factors associated with seeking medical care for depression [13] or the obstacles that could explain the non-seeking of such care, especially in the most vulnerable populations. In France in particular, no research has been carried out to determine the reasons for not seeking primary care during a depressive episode. To investigate this matter, we decided to carry out a two-phase study by conducting a qualitative survey in the general population then a quantitative survey at the Baudelaire Outpatient Clinic at the Saint-Antoine Hospital in Paris. This clinic provides free health and social care to people in precarious situations (mainly individuals with no health insurance, undocumented individuals and/or individuals who are too poor to consult a general practitioner (GP) in the usual health-care system). At or through this clinic, patients can consult a GP or a specialist, obtain social care and free medications, avail themselves of the hospital’s technical services, and be hospitalized, if necessary. Our ultimate objective was to determine the reasons for not seeking primary care during a depressive episode in this vulnerable patient population.

## Methods

Since little is known about the factors associated with seeking or not seeking care for depression, we chose, in order to achieve our ultimate objective, a mixed study with an exploratory sequential design [14]. First, a qualitative design was used to study people’s attitudes and views regarding depression. We used a phenomenological approach (the study of a phenomenon whose structure is determined from a direct analysis of a given individual’s experience), since our aim was to study their actual experiences [15]. We then used a quantitative cross-sectional design to study the reasons for not seeing a GP for depression among patients who visited the clinic. A purposive sampling procedure was used for the qualitative study in order to collect a wide range of opinions with diversified sampling in terms of gender, age and socioeconomic status. Recruitment was done in general practice settings, mother-child health care centers, and in the public transit system. Semidirected, face-to-face interviews were carried out until data saturation between June and August 2010 in Paris and its suburbs. After 30 interviews, no new information emerged. The interviews were recorded and transcribed. We performed a thematic analysis within a phenomenological approach, focusing on the perceptions and the management of depression, and the reasons for not consulting a GP during a depressive episode. The transcripts were open-coded to reach a consensus definition of the categories and subcategories. This coding was done independently by two researchers (CR and PC) for triangulation. An axial coding framework was then developed by reorganizing the open codes. A consensus was reached for each emerging category. A third researcher (IP) reviewed the categories when a consensus was not reached. Second, a cross-sectional observational study was performed among the sample of patients visiting the Baudelaire Outpatient Clinic at the Saint-Antoine Hospital in Paris. Patients seeing a physician for any reason on one of the designated weekdays from September 2010 to December 2010 were asked if they would agree to participate in a study consisting of a face-to-face questionnaire right before their appointment. The answers to the questionnaire were anonymized and confidential and were not given to the clinic’s health professionals.

Since this survey did not fall into the category of biomedical research (as defined by French law) and did not involve collecting any personally identifiable data, it did not require ethical approval in France at the time of the study and only the participants’ oral consent was obtained [16]. This survey was conducted under the ethical responsibility of this free clinic’s chief physician (JL).

A questionnaire was constructed on the basis of the four main categories that emerged from the qualitative analysis. It contained 62 closed-ended questions and collected the following information and data: the patients’ main sociodemographic characteristics, their responses to the questions in the Mini-International Neuropsychiatric Interview (MINI) [17], whether or not they were afraid to talk to the physician about their depression, the type of care sought during a depressive episode (past, present and hypothetical), and the possible reasons for not seeing a GP.

The sociodemographic data included age, sex, nationality, job category status, family status, type of health insurance, cohabitation, educational level, and having or not having declared a regular GP to the Social Security health insurance system.

We used the set of 10 questions in the MINI to identify depressive symptoms during the two weeks preceding the interview. This is a short, structured diagnostic interview based on DSM IV criteria and designed in such a manner that it can be administered by nonspecialist interviewers. Its internal and external validity have been demonstrated in the French population [17]. Using the usual cut-off score (more than three positive responses), which has been validated, we created a binary variable indicating the presence or absence of a current major depressive episode. The individuals who were not depressed at the time of the interview were asked about the occurrence of depressive events in the past (but not about the nature of such episodes, if any).

Next, all the individuals who were experiencing a major depressive episode at the time of the survey and those who had experienced depression in the past were asked about care-seeking during their episodes. For the purposes of the analysis of care-seeking, the interviewees were asked whether they had talked to a physician about their depressive symptoms (about a past or the present episode) and whether they were afraid to talk to their GP about them.

All the interviewees were then asked the following question: When you are or were depressed, or if you became depressed, for what reasons do you, did you or would you not talk to your doctor about it? (with regard to a past, the present or a hypothetical episode). They were given a list of 31 possible reasons. The list had been constructed around the four dimensions identified during the qualitative analysis, and the reasons had been recoded to “Yes” or “No”. Lastly, the patients were asked their opinion about the interest their doctor had shown in their psychological well-being: “During your visits, did your doctor ask about your mood?”.

The chi-square test (or the Fisher’s exact test for small samples) was used for comparisons of proportions. The statistical analyses were performed using Stata v.10 software (StataCorp LP, College Station, Texas, USA).

All data are available on https://mynotebook.labarchives.com/share/claire/MjAuOHw1OTcxMi8xNi0yL1RyZWVOb2RlLzI0MjMwMTAwODd8NTIuOA==.

## Results

### Results of the qualitative analysis

Of the 30 interviewees, 17 were women and 13 were men, and 21 of them were experiencing a depressive episode or had experienced one in the past. All the interviewees gave their perceptions and experiences regarding depression and its treatment. They also indicated the reasons why they might hesitate to talk to their doctor about their depressive symptoms. We will discuss here only those reasons. The investigators identified four main dimensions (Table 1). They are, in order of frequency: the perception of the treatment of depression (n = 29), the perception of depression (n = 26), the social environment (n = 20), and the doctor-patient relationship (n = 14). Each dimension contains a varying number of themes.

**Table 1:** **Dimensions and themes concerning the non-seeking of medical care during a depressive episode as revealed by the qualitative analysis**

**Dimension**	**n**	**Coded types**
**1. Treatment of depression**	**29**	
a) Reluctance:		
- The inefficacy of drugs.	5	“when stop the treatment, you become ill again” (1.21) “to heal, just talk about it. medications don’t work” (7.18.22)
- The possibility that the physician would prescribe drugs with side effects.	2	“fear that the GP will give me medications” (10.30)
- Afraid of the duration and complexity of the treatment.	3	“fear of embarking on a long and complicated treatment” (5.21.25)
- Afraid of the side effects of drugs.	9	“medications aren’t good for your health; they don’t help you feel better” (4.5.6.11.14.15.27)
		“medications are dangerous, especially if you’re young” (13)
		“medications aren’t good for your health. they cause dependencies” (11.13.15.24)
- Afraid to face reality.	2	“I might not want to hear things about my life, so I don’t talk about them” (10) “sometimes, it’s best not to try to find out why you’re not doing well” (10. 11)
b) Psychiatrist better suited than a GP.	5	“I prefer to get the appropriate treatment” (1.3.4)
		“it can be treated with psychotherapy. no medications” (1.20.29)
		“psychiatrists are better suited than GPs” (1.29)
c) Trust in God:		
- God can heal.	3	“I prefer to trust in God for healing” (6.12.23)
- Prayer can heal.	2	“the best thing for healing is prayer” (12.19)
d) Not believing that treatment is necessary:		
- I know why I’m depressed, so if I solve the problem, I’ll feel better.	19	“always a reason” (1.2.4.5.6.7.8.23) “depends on your life events” (2.4.6.7.9.12.14.15.18.27)
		“all you have to do is solve your problems” (1.5.7.14.16.22.26) “just fatigue or working too much” (8.20.27)
- I don’t think I’m depressed (enough).	8	“being in denial” (7.10.13.19.21.24)
		“a bit depressed”(1.16.24) “temporary depression” (1.10.16)
**2. Perception of depression**	**26**	
a) Loss of self-worth (poor self-image):		
- Shame of being depressed.	9	“shame” (3.8.10.11.13.15.17.28.30)
- Feeling guilty towards society.	5	“I feel guilty towards others about being depressed” (2.19.22.25.28)
- Depression is a weakness.	7	“being depressed is being weak” (8.9.14.15) “being depressed is being weak” (7.8.19)
- Lack of virility*.	2	“men can’t be depressed” (8.24) “It’s a virility problem” (8)
b) Stigmatization:		
- Afraid of being judged by society.	3	“afraid of being judged by others” (5.17.30)
- Afraid of being considered crazy.	3	“afraid of being considered crazy by others” (13.17.28)
- I’m afraid of being categorized by society or my friends and family.	4	“when you’re depressed, people stop talking to you” (2.8.17.29)
c) Mental health problems are not diseases.	13	“not an illness” (1.4.7.8.9.11.12.14.15.23.26) “just some malaise” (2.7.18)
d) Depression is a taboo subject.	4	“taboo subject” (8.17) “people don’t talk about mental illnesses” (10.28)
e) I have a family history of depression and don’t want to follow suit.	5	“I know people in my family who are depressed, and I don’t want to be like them” (9.13.16.25) “my mother suffered from depression, and I don’t want to be like her” (5)
**3. Social environment**	**20**	
a) Someone at home to talk to.	12	“I have good support at home” (1.2.8.18) “it gets healed with help from the people around you” (1.3.14) “family needed for healing” (4.11.16.18.26) “you just need to talk to your friends or family” (2.7.10.11.14.26)
b) Afraid to bother those around him/her.	4	“I don’t want to bother those around me with this problem” (2.13.24.27)
c) Incompatibility with social status:		
- Prohibited because of social status.	4	“a high social status means you can’t be depressed” (8.29) “I project to others the image of someone who’s strong” (9.15)
- Prohibited because of role among family and friends.	2	“friends and family distance themselves from depressed people” (5.19)
**4. Doctor-patient relationship**	**14**	
a) Not knowing a doctor to talk to		
- No GP to talk to.	2	“I’ve never had a doctor I could talk to about it” (5.11)
- Don’t know who to talk to.	2	“I don’t know who to talk to about it” (2.8) “I didn’t know that I could talk to my doctor about it” (2.8)
- Don’t know GP well enough.	4	“I don’t have a doctor who knows me well enough to talk about it” (1.17.22.27)
b) Neglect on the part of the GP		
- The GP wouldn’t listen to the complaint	3	“my doctor couldn’t care less about psychological problems” (24.26) “he doesn’t’ listen to me when I talk to him about my mood” (2)
- Bad experiences with GP	1	“I’ve had bad experiences with doctors” (2)
c) Not a GP’s role		
- The GP could not look after this problem.	3	“Doctors tend to real illnesses, not psychological problems” (8.16.24)
- Afraid to bother GP.	8	“I don’t’ want to bother my doctor with this minor problem” (1.2.16.18.20.24.26.28)

#### Perceptions of the treatment of depression

The study identified four themes in this dimension (Table 1): the reluctance to be treated (pharmacological treatment and/or psychotherapy), the preference to see a psychiatrist directly rather than their GP, providential healing, and not believing that treatment is necessary. We chose to classify the last theme in this dimension instead of among perceptions of depression because we believed that it has a direct impact on the management of this disease. The negative perception of the treatments (side effects, inefficacy, complexity, and the duration of treatment) is one of the main impediments identified in our analysis. It is interesting to note that more than half of the interviewees believed that there is a nonmedical etiology to the occurrence of a depressive episode and that it is therefore simply a matter of solving these problems in order to “get out of” one’s depression.

#### Perceptions of depression

Among the reasons cited most frequently for not talking to one’s GP about depressive symptoms were different themes pertaining to the perceptions of depression. Five such themes were identified: a loss of self-worth, stigmatization, the fact that psychological problems are not diseases, the taboo nature of depression, and identifying with family members who suffer from depression. For 13 of the 30 interviewees, depression was not a full-fledged disease, but rather a temporary “malaise” that does not require a visit to one’s doctor. For others (a third of the interviewees), depression was a shameful disease, and for some (7 of them), it was even the reflection of a weakness or even (for 2 men) the reflection of a lack of virility. The social exclusion caused by depression, whether as such (withdrawal and apathy), whether caused by how society views this disease, or whether it was due to the shame the participants felt based on their own values, is another reason reported for not talking to their GP about their depression. A last theme concerns the fear of being like family members known to have depression. They did not want to be like them, many of whom had severe depression. The disease evoked this painful experience. It is, in any event, about remaining undiagnosed, as if disability, shame and fear are bearable as long as the medical profession has not put a name to the illness.

#### The social environment

Three themes were identified in this dimension: having someone close to talk to, being afraid to bother friends and family, and incompatibility with social status. The importance of social support was mentioned by most of the interviewees. For some of them, having moral support at home or the mere fact of talking about their depression with those around them could be enough for healing. On the other hand, some of the interviewees concealed their depression from their families so as not to bother them, out of fear of being rejected or because their social status or the image that others had of them (or the image they liked to project of themselves?) seemed, to them, to be incompatible with revealing their depression. These chosen or forced concealments were all reasons given by the interviewees for not discussing these problems with their GP.

#### The doctor-patient relationship

Three themes were identified in this dimension: not having a GP to talk to about their depression (some of the interviewees indicated that they were not close enough to their GP to confide in him/her), past negative experiences with their GP (reported by one interviewee), and the fact that these problems were outside the scope of their GP’s practice (either because his/she did not treat psychological problems or because the individuals considered these problems too minor for them to seek medical attention).

### Results of the quantitative survey

On the 16 eligible days of the 4-month survey, 255 French-speaking patients visited the clinic. Two of them were excluded because of behavioral disorders incompatible with the administration of a questionnaire (one had acute psychiatric disorders, and the other was inebriated), and three refused to participate. The final sample therefore consisted of 250 patients, for a participation rate of 98.8%.

#### Sociodemographic characteristics

The study participants (mean age: 45 years; 52.0% male) were generally of low socioeconomic status (Table 2). Half of them were foreign immigrants (52.4%). A third (31.2%) had a high level of education, but only 40.0% had a job on the day of the survey (9.6% held managerial/professional positions), 10.0% were unemployed and 16.0% were retired. Half of the participants (55.2%) had the usual health insurance enjoyed by most people in France (i.e., basic insurance under the Social Security system with supplemental insurance), 20.0% were covered by the public insurance plan intended for the poor (CMU; Universal Medical Insurance), 18.8% were insured under a specific government-run procedure for undocumented immigrants (AME; State Medical Aid), and 6.0% were uninsured. In all, more than a third (37.6%) had no supplemental insurance (and were therefore only partially covered by the basic Social Security insurance, being responsible for approximately 30% of their out-of-pocket expenses).

**Table 2:** **Characteristics of the quantitative survey population**

	**Total**		**Men**		**Women**	
	**N**	**%**	**n**	**%**	**n**	**%**
Age						
Under 25	20	8.0	10	7.8	10	8.3
25 to 44	118	47.2	68	52.7	50	41.3
45 to 59	62	24.8	27	20.9	35	28.9
Over 60	50	20.0	24	18.6	26	21.5
**Nationality**						
French	118	47.2	51	39.5	67	55.4
Foreign	132	52.8	78	60.5	54	44.6
**Job category**						
Managerial/professional	24	9.6	8	6.2	16	13.2
Other	225	90.4	120	93.0	105	86.8
**Family status**						
Single	108	43.2	62	48.1	46	38.0
Married or living with partner	109	43.6	55	42.6	54	44.6
Divorced	22	8.8	9	7.0	13	10.7
Widow/widower	11	4.4	3	2.3	8	6.6
**Health insurance**						
Standard	138	55.2	60	46.5	78	64.5
Universal	50	20.0	30	23.3	20	16.5
State medical aid	47	18.8	31	24.0	16	13.2
None	15	6.0	8	6.2	7	5.8
**Cohabitation**						
Family	145	58.0	63	48.8	82	67.8
Non-relative	24	9.6	16	12.4	8	6.6
Living alone	80	32.0	49	38.0	31	25.6
**Regular physician**						
Yes	205	82.0	93	72.1	112	92.6
No	45	18.0	36	27.9	9	7.4
**Educational level**						
High school or less	172	68.8	95	73.6	77	63.6
Higher education	78	31.2	34	26.4	44	36.4
**Feeling of isolation**						
Very alone	28	11.2	18	14.0	10	8.3
Rather alone	84	33.6	49	38.0	35	28.9
Supported	108	43.2	46	35.7	62	51.2
Strongly supported	30	12.0	16	12.4	14	11.6
**Religious beliefs**						
Yes	160	65.6	78	60.5	82	67.8
No	84	34.4	48	37.2	36	29.8

Close to half (43.2%) of the participants reported that they were married or living with someone, and 43.6% were single. However, only 32.0% of the interviewees reported living alone, while 58.0% were living with a family member, and close to 9.6% were living with a non-relative. Lastly, 82.0% reported having a gatekeeping GP.

#### Medical care-seeking during a depressive episode

The prevalence of current major depressive episodes was 56.7%. Of the 144 patients diagnosed with depression during the interviews, only 52.8% reported having talked to a physician about their depression (60.0% of the depressed women and 44.9% of the depressed men; p = 0.07). Only 44.3% of the depressed foreigners reported having done so versus 66.1% of the French nationals (p = 0.01). Also, close to 60% of the participants with no supplemental health insurance had not spoken to a physician compared to 39.2% of those with supplemental insurance and 33.3% of those with CMU (p = 0.02).

#### Reasons for not consulting a GP during a depressive episode

In this section, we provide the frequencies of answers to the question mentioned above (When you are or were depressed, or if you became depressed, for what reasons do you, did you or would you not talk to your GP about it?) for the total number of interviewees. Indeed, there were no significant differences in these frequencies between the three subpopulations (depressed at the time of the survey, not depressed at the time of the survey but with a past history of depression, and never depressed but asked about a hypothetical episode), which enables us to report overall figures.

As in the qualitative survey, a negative perception of depression was one of the most frequent reasons for never seeing a GP (Table 3). Indeed, half of the participants (52.0%) agreed with the fact that they were afraid of being judged negatively by society. A fourth of the interviewees (23.0%) indicated that being depressed made them feel guilty towards society. This feeling was reported more frequently by men (29.0%) than women (17.0%; p = 0.034) and by foreigners (31.1%) than French nationals (14.4%; p = 0.002). A majority of the participants indicated that they did not need to see a doctor because they knew why they were depressed (75.0%) or that they were in denial (68.0%). For many men (37.2%), depression was not perceived as a disease (versus 19.0% of the women; p < 0.001). For a fifth of the foreigners (20.5%), being depressed was a problem of virility (versus 8.5% of the French nationals; p = 0.04).

**Table 3:** **Proportions of respondents citing reasons for not consulting a GP for depression, by gender and nationality**

	**Total (n = 250)**		**Men (n = 129)**		**Women (n = 121)**			**Foreigners (n = 132)**		**French (n = 118)**		
	**N**	**%**	**n**	**%**	**n**	**%**	**p**	**n**	**%**	**n**	**%**	**p**
**1. Treatment of depression**												
a) Reluctance:	229*	91.6	121*	93.8	108*	89.3	0.196	120*	90.9	109*	92.4	0.677
- The inefficacy of drugs.	137	54.8	69	53.5	68	56.2	0.667	61	46.2	76	64.4	0.004
- The possibility that the physician would prescribe drugs with side effects.	173	69.2	84	65.1	89	73.6	0.149	87	65.9	86	72.9	0.233
- Afraid of the duration and complexity of the treatment.	136	54.4	74	57.4	62	51.2	0.331	71	53.8	65	55.1	0.837
- Afraid of the side effects of drugs.	142	56.8	73	56.6	69	57.0	0.945	58	43.9	84	71.2	<0.001
- Afraid to face reality.	121	48.4	59	45.7	62	51.2	0.384	75	56.8	46	39.0	0.005
b) Psychiatrist better suited than a GP	76	30.4	40	31.0	36	29.8	0.829	34	25.8	42	35.6	0.091
c) Trust in God:	173*	69.2	85*	65.9	88*	72.7	0.242	108*	81.8	65*	55.1	<0.001
- God can heal	166	66.4	81	62.8	85	70.2	0.212	103	78.0	63	53.4	<0.001
- Prayer can heal	158	63.2	78	60.5	80	66.1	0.355	99	75.0	59	50.0	<0.001
d) Not believing that treatment is necessary:	200*	80.0	99*	76.7	101*	83.5	0.184	102*	77.3	98*	83.1	0.254
- I know why I’m depressed, so if I solve the problem, I’ll feel better	188	75.2	94	72.9	94	77.7	0.378	97	73.5	91	77.1	0.507
- I don’t think I’m depressed (enough)	170	68.0	84	65.1	86	71.1	0.313	84	63.6	86	72.9	0.118
**2. Perception of depression**												
a) Loss of self-worth (poor self-image):	169*	67.6	89*	69.0	80*	66.1	0.627	102*	77.3	67*	56.8	0.001
- Shame of being depressed .	90	36.0	48	37.2	42	34.7	0.681	56	42.4	34	28.8	0.025
- Feeling guilty towards society.	58	23.2	37	28.7	21	17.4	0.034	41	31.1	17	14.4	0.002
- Depression is a weakness.	129	51.6	72	55.8	57	47.1	0.169	77	58.3	52	44.1	0.024
- Lack of virility*.	37	28.7**	37	28.7				27	20.5	10	8.5	0.040
b) Stigmatization:	75*	70.0	88*	68.2	87*	71.9	0.525	97*	73.5	78*	66.1	0.203
- Afraid of being judged by society.	101	40.4	56	43.4	45	37.2	0.316	64	48.5	37	31.4	0.006
- Afraid of being considered crazy.	98	39.2	44	34.1	54	44.6	0.089	54	40.9	44	37.3	0.558
- I’m afraid of being categorized by society or my friends and family.	130	52.0	63	48.8	67	55.4	0.301	74	56.1	56	47.5	0.174
c) Mental health problems are not diseases.	71	28.4	48	37.2	23	19.0	<0.001	44	33.3	27	22.9	0.067
d) Depression is a taboo subject.	95	38.0	65	50.4	30	24.8	<0.001	56	42.4	39	33.1	0.127
e) I have a family history of depression and don’t want to follow suit.	71	28.4	35	27.1	36	29.8	0.646	34	25.8	37	31.4	0.327
**3. Social environment**												
a) Someone at home to talk to.	175	70.0	90	69.8	85	70.2	0.934	88	66.7	87	73.7	0.224
b) Afraid to bother those around him/her.	147	58.8	77	59.7	70	57.9	0.768	73	55.3	74	62.7	0.235
c) Incompatibility with social status:	99*	39.6	57*	44.2	42*	34.7	0.126	56*	42.4	43*	36.4	0.334
- Prohibited because of social status.	76	30.4	44	34.1	32	26.4	0.188	45	34.1	31	26.3	0.180
- Prohibited because of role among family and friends.	71	28.4	40	31.0	31	25.6	0.345	42	31.8	29	24.6	0.205
**4. Doctor-patient relationship**												
a) Not knowing a doctor to talk to	125*	50.0	74*	57.4	51*	42.1	0.016	73*	55.3	52*	44.1	0.076
- No GP to talk to about it.	83	33.2	45	34.9	38	31.4	0.559	57	43.2	26	22.0	<0.001
- Don’t know who to talk to.	96	38.4	57	44.2	39	32.2	0.052	60	45.5	36	30.5	0.015
- Don’t know GP well enough.	108	43.2	66	51.2	42	34.7	0.009	69	52.3	39	33.1	0.002
b) Neglect on the part of the GP	112*	44.8	63*	48.8	49*	40.5	0.185	60*	45.5	52*	44.1	0.826
- The GP would not listen to the complaint	110	44.0	54	41.9	56	46.3	0.482	52	39.4	58	49.2	0.121
- Bad experiences with GP.	60	24.0	38	29.5	22	18.2	0.037	37	28.0	23	19.5	0.115
c) Not a GP’s role	158*	63.2	81*	62.8	77*	63.6	0.890	81*	61.4	77*	65.3	0.524
- The GP could not look after this problem.	88	35.2	47	36.4	41	33.9	0.673	43	32.6	45	38.1	0.358
- Afraid to bother GP.	101	40.4	51	39.5	50	41.3	0.773	56	42.4	45	38.1	0.490

*At least one event.

On the subject of depression treatments, nearly 70% of the interviewees indicated that they were afraid of the side effects of the drugs used to treat depression. Two-thirds of the French participants (64.0%) thought that drugs were ineffective in curing depression. This belief was less prevalent among the foreigners (46.2%; p = 0.004). A majority of the interviewees (over 60%) stated that God or prayer could help in healing, with more foreigners (over 75% of them gave this as a reason for not seeing a GP) stating this than French interviewees (50.0%; p < 0.001).

As regards friends and family, 70.0% of the participants indicated that having someone at home to talk to was enough for healing, with no differences between the men and women or between the foreigners and French nationals. However, 59.0% of the interviewees said that they were afraid to bother those around them with this problem, a finding consistent with the qualitative survey.

Finally, with regard to the doctor-patient relationship, 8.0% stated that they were afraid to talk to their doctor about their depression. Nearly half of the interviewees (44.0%) did not do so because they thought that their physician did not know them well enough. This reason was reported more frequently by the men (51.0%) than the women (35.0%; p = 0.009) and by the foreigners (52.0%; p = 0.002) than the French nationals. Almost half of the interviewees thought that their GP would not be attentive to their complaint, and more than a third (40.0%) were afraid to bother him/her with this problem.

#### Impact of depression history

Table 4 shows the results in terms of the history of depression. It is seen that some reasons are associated with no care-seeking more often among the participants who had a current or past history of depression than among those interviewed about a hypothetical episode. For example, a number of reasons concern the perception of depression, such as shame of being depressed (42.6% for the participants with a current episode, 15.5% for those with a previous history of depression and 22.1% for the participants interviewed about a hypothetical depressive episode; p = 0.016) and the fear of being judged by society (48.8%, 35.8% and 27.9%, respectively; p = 0.013). Others concern the experience of the disease or its treatments. A poor doctor-patient relationship was cited more often by those with a history of depression than by those interviewed about a hypothetical depressive episode.

**Table 4:** **Impact of history of depression on reasons for not seeking care during depression**

	**Current episode (n = 129)**		**Past episode (n = 53)**		**Hypothetical episode (n = 68)**		
	**n**	**%**	**n**	**%**	**n**	**%**	**p**
**1. Treatment of depression**							
a) Reluctance:							
- The inefficacy of drugs.	73	56.6	30	56.6	34	50.0	0.648
- The possibility that the physician would prescribe drugs with side effects.	76	58.9	31	58.5	35	51.5	0.582
- Afraid of the duration and complexity of the treatment.	70	54.3	31	58.5	35	51.5	0.743
- Afraid of the side effects of drugs.	98	76.0	34	64.2	41	60.3	0.051
- Afraid to face reality.							
b) Psychiatrist better suited than a GP	40	31.0	18	14.0	18	26.5	0.658
c) Trust in God:							
- God can heal	89	69.0	29	54.7	48	70.6	0.125
- Prayer can heal	84	65.1	29	54.7	45	66.2	0.349
d) Not believing that treatment is necessary:							
- I know why I’m depressed. so if I solve the problem. I’ll feel better	98	76.0	40	31.0	50	73.5	0.93
- I don’t think I’m depressed (enough)	92	71.3	32	24.8	46	67.6	0.355
**2. Perception of depression**							
a) Loss of self-worth (poor self-image):							
- Shame of being depressed.	55	42.6	20	15.5	15	22.1	0.016
- Feeling guilty towards society.	39	30.2	6	4.7	13	19.1	0.015
- Depression is a weakness.	71	55.0	25	19.4	33	48.5	0.526
- Lack of virility*.	26	20.2	1	0.8	10	14.7	0.002
b) Stigmatization:							
- Afraid of being judged by society.	63	48.8	19	35.8	19	27.9	0.013
- Afraid of being considered crazy.	59	45.7	16	30.2	23	33.8	0.084
- I’m afraid of being categorized by society or my friends and family.	85	65.9	22	41.5	23	33.8	<0.001
c) Mental health problems are not diseases.	36	27.9	12	22.6	23	33.8	0.394
d) Depression is a taboo subject.	55	42.6	16	30.2	24	35.3	0.252
e) I have a family history of depression and don’t want to follow suit.	37	28.7	17	32.1	17	25.0	0.689
**3. Social environment**							
a) Someone at home to talk to.	93	72.1	33	62.3	49	72.1	0.384
b) Afraid to bother those around him/her.	82	63.6	31	58.5	34	50.0	0.184
c) Incompatibility with social status:							
- Prohibited because of social status.	40	31.0	12	22.6	24	35.3	0.317
- Prohibited because of role among family and friends.	43	33.3	11	20.8	17	25.0	0.178
**4. Doctor-patient relationship**							
a) Not knowing a doctor to talk to							
- No GP to talk to about it.	52	40.3	14	26.4	17	25.0	0.047
- Don’t know who to talk to.	55	42.6	17	32.1	24	35.3	0.341
- Don’t know GP well enough.	66	51.2	21	39.6	21	30.9	0.02
b) Neglect on the part of the GP							
- The GP would not listen to the complaint	53	41.1	17	32.1	18	26.5	0.108
- Bad experiences with GP.	36	27.9	15	28.3	9	13.2	0.051
c) Not a GP’s role							
- The GP could not look after this problem.	61	47.3	22	41.5	27	39.7	0.547
- Afraid to bother GP.	62	48.1	18	34.0	21	30.9	0.037

*Men only were interviewed about this proposition.

## Discussion

The objective of our study was to identify the reasons for which socially vulnerable patients are reluctant to talk to their GP about their depressive symptoms and to determine the sociodemographic characteristics associated with the different reasons for not consulting a GP. We used a mixed method combining a qualitative and a quantitative approach. Our study found that the reasons for not seeing a GP can be grouped into four main dimensions: the negative perception of the disease, the negative perception of its treatment, the importance of the social environment, and the doctor-patient relationship.

Our study has some limitations. First, our sample size for the quantitative survey was rather small. Second, we observed a high prevalence of depression at the time of the survey, which may have affected our results. Indeed we observed some differences between the (then or previously) depressed and nondepressed patients with regard to the reasons given for not consulting a GP: Such as the perception of shame, the fear of being judged by society and being afraid to bother their GP (each of which were cited more often by the depressed patients than the nondepressed ones, who were interviewed about a hypothetical situation), other significant differences could, of course, have been found with a larger sample, but none were observed in our study. In addition, we did not observe any significant differences regarding the impact of the social environment. Finally, since this study concerned vulnerable individuals with a very low socioeconomic status who visited a free clinic, our results cannot obviously be extrapolated to the general population, but this was not our objective. We also could have conducted the qualitative study in the same area as the setting for the quantitative study, as recommended in a mixed-method protocol [14]. However, we chose to achieve maximum diversity among participants from the general population to better understand their views and experiences for the purpose of the second, i.e., quantitative, survey. We did think that there could be a selection bias if we studied only participants visiting the clinic. We also used the evaluation document for mixed methods developed by Tong in order to design our qualitative sample [18]. Lastly, the individuals who were not depressed at the time of the interview were asked about past depressive episodes but not about their nature or form. This decision may have led to underreporting bias.

It appears that close to 50% of the depressed individuals did not seek care during their depression. It is known that all care-seeking depends on the pain level, but not in a linear fashion. In fact, in major depressive disorder, the suffering can be so intense that the individual persists in not believing in treatments, but at the same time he/she must experience a certain level of suffering to consider his/her depression a clinical problem [19]. It seems that women see a GP more often than men during a depressive episode and that people of French nationality do so more often than foreigners. The fact that men and people from ethnic minorities see a GP less often for psychiatric problems is well documented in the United States [20-22] but much less so in France. Indeed, it has been widely described that, in France, immigrants use health services less than French nationals on average, whether primary or specialized care [23,24], but the reasons for not consulting a GP have been systematically explored to a far lesser extent, particularly in the area of mental health and more specifically in cases of depression.

Negative attitudes toward and perceptions of depression were the most common reasons for not seeing a GP. This disease still seemed taboo or shameful for many of the participants. Men and foreigners expressed feelings of stigma more often whether such feelings were directed towards them and/or from them towards others. Past studies have shown that racial and ethnic minorities, and especially adolescents in these minorities, may be more susceptible to the impact of stigma from having and being treated for a psychiatric disorder [25].

Moreover, a meta-analysis published in 2007 suggests that patient adherence was positively correlated with their beliefs about the seriousness or severity of the disease to be prevented or treated [26]. We can assume that the level of willingness to consult a doctor may obviously be low when depression is not perceived as a serious disease but rather as a temporary mood problem, possibly due to external and reversible factors. In certain previous studies on depression in primary care, the authors observed that when some patients believed they had the “social type” of depression, they were more frequently reluctant to accept medical treatment [27-29].

It is a different situation when people believe that God or prayer can help in healing or when they prefer to turn to informal supports rather than traditional mental health services, as described in certain racial and ethnic minorities [30]. In such cases, it seems that it is health professionals’ abilities or even the legitimacy of modern medicine itself that are or is questioned. Moreover, whether it is considered a disease or not, depression is not perceived as one of a physician’s area of competence. This point is supported by the study of Ng et al. [31], who found that elderly Singaporeans of all religious affiliations―a population with a higher prevalence of mental health problems than those with no religious affiliation―reported the lowest health professional consultation rate. This differs from studies in Western countries that found no significant differences in mental health services utilization by the elderly based on their religious affiliation or participation [32,33]. Of course, faith and religion may also have a direct impact on mental health. Religious faith may buttress one’s own sense of control and self-esteem [31,33-35], and it reduces anxiety and provides hope [36]. In addition, religious participation widens the network for social integration and support [37], enhances the individual’s sense of security and facilitates his/her adjustment to stress [31,38]. For all these reasons, religious participation can contribute to less use of psychotherapy or pharmacological therapy.

In other respects, theory and research in psychiatry and related mental health fields have challenged negative stereotypes of religion and led to a more nuanced view that recognizes the double-edged capacity of religion to foster both problems and solutions, and distress and relief among people with a serious mental illness [39]. A more differentiated view of religion probably holds significant implications for assessment and treatment [40].

Perceiving depression as a sign of weakness was often mentioned in our qualitative survey and was frequently cited in our quantitative survey as a reason for not seeing a GP. A recent population-based study conducted in Finland [41] did not report this reason for the nonuse of mental health services in cases of depression. Apart from the fact that the study sample was considerably different from ours (52.8% of our sample were foreigners, who mentioned this perception more frequently than the French nationals), this shows how important it is for a quantitative survey to be preceded by a qualitative study in which one systematically proposes reasons that might not emerge from an open-ended question in the quantitative survey.

The negative perception of depression medications was another main theme expressed. The side effects, the duration of treatment, and the inefficacy of these drugs were all reasons given for not consulting a GP, especially, once again, by the foreigners. In their study of treatments for anxiety disorder among 273 women in the United States, Zoellner et al. [42] found that wariness about side effects was one of the reasons the participants were significantly more likely to choose cognitive- behavioral therapy than medication as the treatment for their problem. Similarly, Deacon and Abramawitz in 2009 [43] and Van Geffen et al. [7] in 2010 observed that patients’ perceptions of illnesses and treatments influenced their decisions about taking antidepressants. Another study [9] reports that a majority of the patients considered antidepressants addictive and that a large majority of them indicated that they preferred psychological forms of treatment to medications. The study also notes that the healthy individuals had a more negative perception of pharmacological treatments than the depressed ones. We did not observe this difference in our study between the then or previously depressed patients and those with no history of depression, but we may have lacked statistical power because of the relatively small size of our study population.

The importance of the social environment as a reason for not seeing a GP was frequently cited in our study (both in the qualitative and the quantitative survey). More than two-thirds of the participants said that having someone at home to talk to can help in healing and could be the reason for not consulting a GP during a depressive episode. This finding is supported by the study of Kovess-Masfety et al. [44], in which, compared to women, men mentioned their family and friends more frequently than any care provider. This may reflect a reluctance to accept a mental health care approach. In a previous Australian survey, Griffiths et al. [45] interviewed people about the perceived advantages and disadvantages of seeking help for depression from family and friends. The most commonly cited advantage was social support, including emotional support, while the most commonly cited disadvantage was stigma. Social support is generally seen as and observed to be a a factor prompting the use of health-care services [45-47], including preventive services [48,49]. We have not found any study that specifically examines the impact of social support on seeing a GP for depression, but to our knowledge, our study is the first to find that social support can result in a person not consulting a doctor. It is noteworthy that this reason was cited at a similar frequency by the patients with and without a personal history of depression (62.5% and 55.6%, respectively) and in all our analysis subgroups.

The last category of reasons for not seeing a GP concerns the characteristics of the doctor-patient relationship. Close to 10% of the patients said that they would be afraid to talk to their doctor about their depression. A similar figure for fear was reported in another French survey among a comparable sample of vulnerable patients who visited free clinics in 2000 [50]. In that survey, 12.5% of the patients said that they would be afraid to consult a physician. Men and foreigners indicated more frequently that their GP did not know them well enough for them to entrust him/her with this type of problem, and many of the patients stated that their GP might not listen to their complaints. These findings are consistent with those of Wun et al. who, in another mixed-method study (qualitative and quantitative), found that, for depressed individuals, having a good relationship with their GP was conducive to them consulting him/her first [51]. They noted the importance of the doctor-patient relationship to the interviewees, of the GP having a better knowledge of their case, and of less stigma. In fact, the authors found that the stigma attached to seeing a psychiatrist was a reason for the participants to visit their GP first. In the same French free clinic context, Collet et al. [50] observed that poor and/or underserved patients developed more distrust or defiance toward psychiatrists than their GP.

Although person-centered primary care that is responsive to patients’ goals and preferences is widely recognized as an important and fruitful approach in general practice [52], Olde Hartmann et al. [53] identified the ways in which it can conflict with evidenced-based approaches, which are increasingly valued in practicing, evaluating and regulating primary care. It is possible that these approaches create distance between patients and physicians, with the result that patients think that their doctor is not (or no longer) interested in their “real-life” problems. This would be particularly worrisome in the field of mental health and depression, and even more so at a time when primary care physicians’ workload is heavy. They may think and even seem to patients that they are running out of time to look after their mood disorders. This could explain why, in our sample, some patients said that they did not want to bother their doctor with this “minor problem” and often said that he/she had no time for it. It is noteworthy that this runs counter to patients’ expectations. Indeed, in our survey population, we previously reported that 80% of the interviewees indicated that they would like their GP to ask them about their mood more often, and more than two-thirds said that they would prefer to talk to their GP first about their depression than to a psychiatrist [54], a choice that was also more frequent in a French population-based survey conducted in the Paris region in 2002 [44].

## Conclusions

Considered together, our results argue for better information on depression and its treatments, and for an improvement in its detection by primary care givers, in a more proactive and systematic manner, in poor, vulnerable or underserved patients, particularly among immigrants and in ethnic minorities. Clearly, talking about mood disorders more often and more openly during a primary care consultation in order to destigmatize, diagnose and possibly treat depression requires a trusting relationship between the doctor and patient.

## Abbreviations

GP: General practitioner
MINI: Mini-International neuropsychiatric interview
